# ‘Working relationships’ across difference - a realist review of community engagement with malaria research

**DOI:** 10.12688/wellcomeopenres.17192.1

**Published:** 2022-01-13

**Authors:** Robin Vincent, Bipin Adhikari, Claire Duddy, Emma Richardson, Geoff Wong, James Lavery, Sassy Molyneux, The REAL team: Mary Chambers, Phaik Yeong Cheah, Al Davies, Kate Gooding, Dorcas Kamuya, Vicki Marsh, Noni Mumba, Deborah Nyirenda, and Paulina Tindana.

**Affiliations:** 1 Centre for Tropical Medicine and Global Health, Nuffield Department of Clinical Medicine, University of Oxford, Oxford, OX3 7LG, UK; 2 Robin Vincent Learning and Evaluation Ltd, Sheffield, S89FH, UK; 3 Mahidol-Oxford Tropical Medicine Research Unit, Faculty of Tropical Medicine, Mahidol University, Bangkok, Thailand; 4 Nuffield Department of Primary Health Care Services, University of Oxford, Oxford, OX2 6GG, UK; 5 Health Research, Evidence and Impact, McMaster University, Hamilton, ON L8S 4L8, Canada; 6 Hubert Department of Global Health, Rollins School of Public Health, Emory University, Atlanta, Georgia, 30322, USA; 7 Center for Ethics, Emory University, Atlanta, Georgia, 30322, USA; 8 Kenya Medical Research Institute (KEMRI) Wellcome Trust Research Programme, University of Oxford, Kilifi, 80108, Kenya

**Keywords:** Community Engagement, health research, malaria research, stakeholder engagement, research ethics, realist review, research benefits, access to health

## Abstract

**Background**: Community engagement (CE) is increasingly accepted as a critical aspect of health research, because of its potential to make research more ethical, relevant and well implemented. While CE activities linked to health research have proliferated in Low and Middle Income Countries (LMICs), and are increasingly described in published literature, there is a lack of conceptual clarity around how engagement is understood to ‘work’, and the aims and purposes of engagement are varied and often not made explicit. Ultimately, the evidence base for engagement remains underdeveloped.

**Methods**: To develop explanations for how and why CE with health research contributes to the pattern of outcomes observed in published literature **, **we conducted a realist review of CE with malaria research – a theory driven approach to evidence synthesis.

**Results:** We found that community engagement relies on the development of provisional ‘working relationships’ across differences, primarily of wealth, power and culture. These relationships are rooted in interactions that are experienced as relatively responsive and respectful, and that bring tangible research related benefits. Contextual factors affecting development of working relationships include the facilitating influence of research organisation commitment to and resources for engagement, and constraining factors linked to the prevailing ‘dominant health research paradigm context’, such as: differences of wealth and power between research centres and local populations and health systems; histories of colonialism and vertical health interventions; and external funding and control of health research.

**Conclusions**: The development of working relationships contributes to greater acceptance and participation in research by local stakeholders, who are particularly interested in research related access to health care and other benefits. At the same time, such relationships may involve an accommodation of some ethically problematic characteristics of the dominant health research paradigm, and thereby reproduce this paradigm rather than challenge it with a different logic of collaborative partnership.

## Introduction – community engagement as integral to health research

The disproportionate disease burden in LMICs combined with the predominant focus of research funding on health in high income countries creates an imperative for more high quality, ethical health research in LMICs ( Currat *et al*., 2004; Molyneux *et al*., 2021). Community Engagement (CE) is increasingly accepted as a critical aspect of health research because of its potential to contribute to more ethical, relevant and well implemented research. CE is seen to play an important role in meeting the ethical commitments of research, by supporting adaptation of agreed ethical principles to the varying cultural contexts in which research is conducted ( Adhikari *et al*., 2020; Marsh *et al*., 2008) and potentially extending those commitments to ethics of social justice and solidarity ( Pratt *et al*., 2020a). Community Engagement (CE) in health research has evolved pragmatically with the growth of large research programmes in LMICs, guided by reflexive critique of social scientists and ethicists, and pressure from HIV Social movements in particular ( Slevin *et al*., n.d.). Research ethics guidance often now includes meaningful engagement of research participants at all stages of the research process (see for example CIOMS, 2016; UNAIDS, 2011).

CE has been defined as a process of collaborative work with groups of people affiliated by geographic proximity, interest or health issues, to address social and health challenges affecting those people ( NIH, 2011). Definitions of CE and broader ‘public engagement’ or ‘stakeholder engagement’ are contested, however ( Aggett *et al*., 2012; Participants at CE workshop, 2013), and vary across the domains of health programmes, health research and international development. In our review, CE primarily involved interactions with local research stakeholders directly affected by clinical and public health research initiatives in LMICs, including as potential research participants.

### Aims of community engagement

The purposes of engagement and the way engagement practices are understood to support these purposes in particular research initiatives are not always explicit or clear ( Tindana *et al*., 2007). A distinction is often made between the instrumental goals of CE of improving quality and relevance of research, including improving recruitment targets and a range of ethical goals for CE ( Lavery, 2018). The latter include respecting stakeholders, building relationships, minimising risks, supporting consent processes, understanding vulnerabilities and researcher obligations ( Adhikari *et al*., 2019). In practice however, CE initiatives in health research often have more than one goal, and the distinction between instrumental and ethical goals can be unclear, something which is rarely made explicit in planning or evaluation. This is, in part, due to the fact that ethical negotiation of relationships may be important for achieving more instrumental research goals ( Geissler *et al*., 2008; Participants at CE workshop, 2013) – such as in determining appropriate benefits, supporting consent processes, gaining approvals and building legitimacy for research.

Engagement activities and strategies in health-related research are also diverse. CE encompasses practices including meetings with community members and representatives; information and communication activities to raise awareness and solicit support for research; community advisory boards as a conduit between researchers and local research stakeholders; and involving stakeholders in designing and implementing research activities ( Adhikari *et al*., 2016; Participants at CE workshop, 2013). Calls for more systematic evaluation of engagement have highlighted a need for greater clarity about intended outcomes and understandings of whether and how engagement activities contribute to these outcomes ( Lavery, 2018; MacQueen *et al*., 2015; Newman, 2006; Participants at CE workshop, 2013)

### Addressing complexity

CE displays characteristics typical of complex social processes, including multiple stakeholders with different understandings and interests interacting over time; a diversity of key activities with long implementation chains; and wider cultural and economic influences on people’s decision-making ( Egan *et al*., 2019; Pawson, 2013). CE is also specifically influenced by the economics and politics of health and research, international organisations and funders, and bioethics codes rooted in particular philosophical traditions ( Reynolds & Sariola, 2018). The wider context for health research in LMICs is one where it is increasingly funded and governed by large transnational research organisations and ‘partnerships’ ( Clinton & Sridhar, 2017; Lavery, 2004; Mahajan, 2019) and deployed through an international infrastructure of ‘techno-science’ that mixes public and private provision ( Leach & Fairhead, 2007). Many clinical trials are ‘off-shored’ to LMICs ( Petryna, 2007) where the resources accompanying such trials can be a significant boost for health systems weakened by privatization and reduced social spending instituted under neo-liberal economic arrangements ( Blume, 2017; Ferguson, 2006). Some scholars argue that engagement with research in these contexts can amount to ‘structural coercion’ – given the wider constraints on people’s decision-making ( Farmer, 2004; Kingori, 2015; Nyirenda *et al*., 2020).

This extraordinary complexity of CE means there are a range of influences and dynamics at different levels to confront when planning and implementing CE ( Lavery, 2018). A growing body of empirical work highlights aspects of the more immediate context in which engagement takes place that influence the opportunities and constraints for engagement, including the type of research and intervention being studied, the influence of local cultural and social practices and beliefs, and the local dynamics of authority and politics ( Adhikari *et al*., 2019). Some rich qualitative case studies and empirical work in relatively mature health research programmes also provide more detailed analysis of some of the relational dynamics at stake in CE and highlight the importance of broader structural influences ( Kamuya *et al*., 2013; Marsh *et al*., 2008; Marsh *et al*., 2011; Molyneux *et al*., 2013).

### Conceptual and practical diversity of engagement literature

In addition to the complexity of CE as a social process, a further challenge for reviewing literature to understand the core logic of CE is the diversity of conceptual and practical influences on CE. The focus of our review is CE in biomedical research trials in LMICs with large malaria trials as the entry point. Even for these studies, a wide range of terms - consultation, participation, engagement and involvement – can be used to describe CE. In other contexts, for example where research is undertaken by development programmes in LMICs, and in community development and patient and public involvement in the global north, these terms may be used differently. CE in health research in LMICs sometimes eclectically borrows from these other ‘traditions’ of engagement, drawing on different conceptual underpinnings and accompanying methods without always being explicit.

***Understanding Community Engagement.*** A better understanding of how CE works in practice and the ways it plays out differently in different contexts is important for more systematic planning and evaluation of CE, to ensure research is both as ethical as possible and has the most impact. A realist review approach is well suited to addressing the complexity that is characteristic of CE. It does this through a focus on ‘programme theory’—iteratively refined causal explanations for how CE contributes to observed outcomes, across varying implementation contexts, through key relational mechanisms—drawn from analyses of relevant empirical data. These key features of realist review stand in sharp contrast to systematic reviews, which average effect sizes across more consistently comparable interventions, but tend to leave the dynamics of ‘how change happens’ as a black box ( Pawson, 2006).

To develop explanations for how and why CE with health research contributes to the pattern of outcomes observed in published literature, we conducted a realist review of CE with health research – a theory driven approach to evidence synthesis. Given the diversity and heterogeneity of the literature, we narrowed the main scope of the review to focus on CE in malaria research trials in LMICs. Data were analysed and synthesized using a realist logic of analysis to understand the mechanisms underpinning engagement, drawing on both empirical studies and relevant social theory to give an explanatory account of some of the key dynamics at stake.

Our review lays out the overall complexity of the engagement landscape in greater resolution than before. It brings into clearer view some of the ethical challenges surrounding CE, which have been an enduring concern in discussions of power, participation and equity in health and research governance, and have been given new impetus by recent debates on decolonising global health. Our review also highlights relationships between health research initiatives and the under-resourced health systems in many LMIC research contexts and raises questions about how an integrated approach might strengthen both. In places the review also goes ‘deeper’, and analysis of particular aspects of engagement allows us to develop practical recommendations. However, overall, lack of clarity around the purposes and causal logic of engagement in the literature reviewed, underscores the need for more empirical research, evaluation and critical analysis of CE as an important field of theory and practice.

## Methods

Our review used a realist review approach in line with the RAMESES guidelines ( Wong *et al*., 2014), the key steps of which are summarized in Table 1. A detailed description of the review methods is provided in the published protocol ( Adhikari *et al*., 2019) and more detail of the steps in Appendix 1, with details of the search strategies in Appendix 2. available in the extended data linked to the paper

**Table 1.** steps in the REAL realist review process.

Step 1a: Locating existing theories, development of initial programme theory	• Identified 28 papers that were used to identify key causal dynamics and contextual influences on CE, with input from scholar/practitioner experts. • Initial programme theory developed at inception workshop with core team and context experts.
Step 1b: (iterative throughout) Conceptual resources	• Substantive theories and concepts relevant to our programme theory were drawn on to inform development of Context Mechanism Outcome configurations (CMOCs) – realist causal explanations that combine into an overall programme theory.
Step 2: Searching for evidence	• Our main search focused on malaria research trials. • Potential additional searches on other health research paradigms were not conducted due to the volume of literature available from the main search. • Citation tracking and drawing on ‘sibling’ and ‘kinship’ papers identified additional papers where our programme theory needed strengthening.
Step 3: Document selection	• Documents were screened by BA using titles and abstracts and then by full text. 10% random sample were reviewed by RV. • Full text documents were selected based on their ability to provide relevant data for the review; including relevant empirical detail or explanatory accounts of engagement outcomes.
Step 4: Data extraction	• Selected documents were coded in Nvivo 12 ^1^. • Coding frameworks were developed independently by two reviewers and iteratively refined with input from the team.
Step 5: Data analysis and synthesis	• Working across and within coded data extracts, CMOCs were iteratively developed and refined by two reviewers with input from the wider team.
Step 6: Refine programme theory	• The overall programme theory was iteratively refined and finalised with input from the review team and content expert advisors, including at a ‘validation workshop’ towards the end of the review

## Results

**Figure 1.**
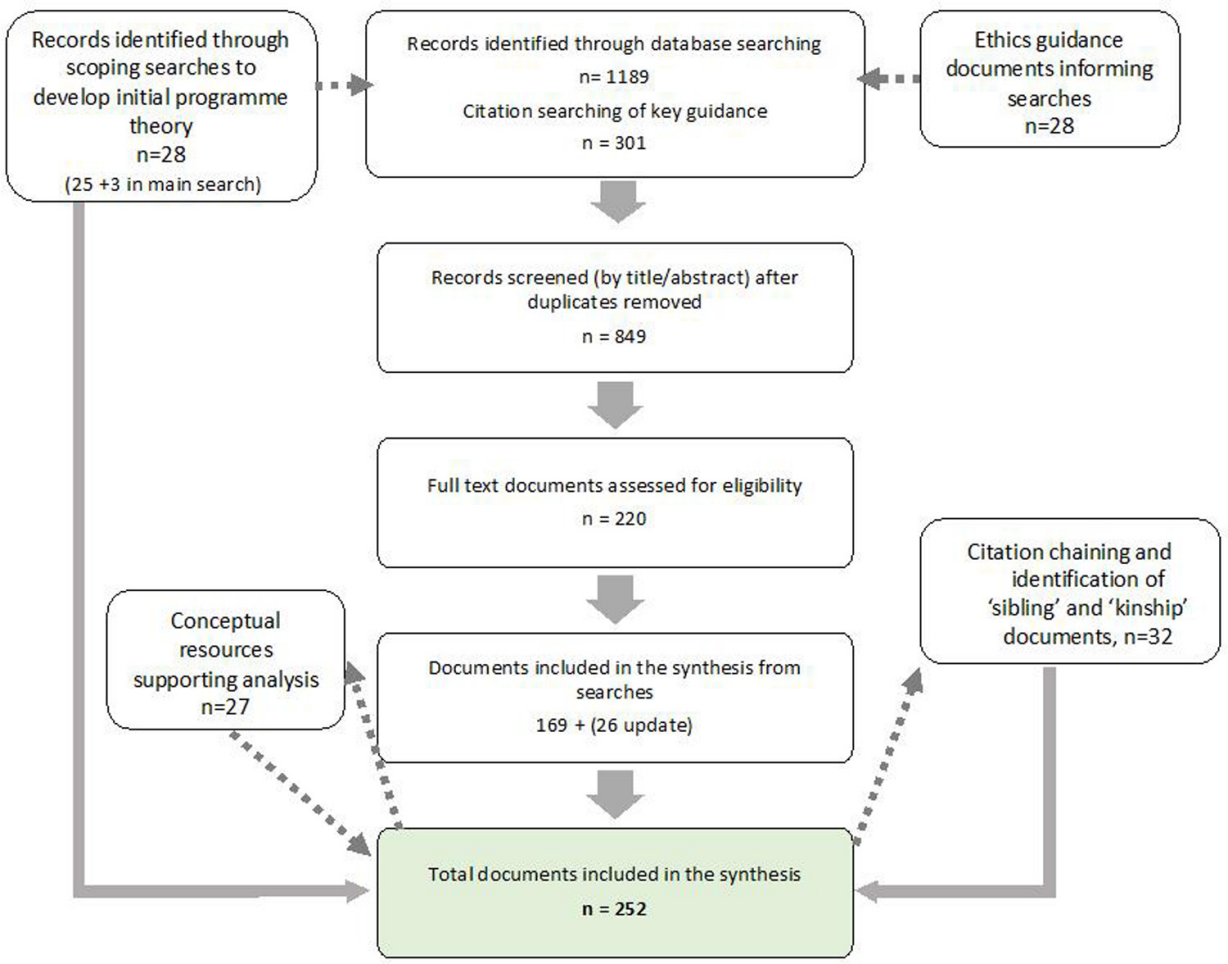
Summary of REAL search process.

Our review identified 252 documents for inclusion in the synthesis. Initial scoping searches with input from thematic experts identified 28 papers (not only focused on malaria trials) that helped us to develop candidate programme theories – initial causal explanations about how CE contributes to observed outcomes. A search of CE in large malaria trials identified 195 documents (3 of which were also identified in the scoping searches). 32 additional papers were identified through citation tracking which provided theoretical insights and empirical data to strengthen our analysis relating to key aspects of the programme theory, including fieldworker intermediary roles, research related benefits, power and constrained agency, accountability in engagement. These were predominantly ‘sibling’ papers (papers providing additional detail on interventions discussed in literature from the main searches), with a handful of ‘kinship’ papers (which addressed related conceptual themes). The overall search process is outline in Figure 1 below.

The literature reviewed primarily came from Africa (147 papers), with 52 papers having a general international focus. There were 28 studies from South East Asia, six from India and six from Central and South America and six from Oceana. Relevant social theories were drawn on to inform development of Context Mechanism Outcome Configurations (CMOCs) - causal propositions explaining how important contexts trigger mechanisms to generate observed outcomes, that appear evident in the selected extracts of data. Appendix 3 in *Extended data* provides a table of document characteristics and how the 252 documents contributed to different CMOCs within the analysis. Appendix 4 in *Extended data* gives details of the main conceptual resources and social theories that we drew on in order to develop CMOCs.

### Descriptive and a-theoretical character of much of the literature

Our review focus on large malaria trials potentially reflects ‘common current practice’ of much biomedical research in LMICs. Accounts of engagement in this literature are often descriptive and a-theoretical, tending to be an adjunct to more detailed accounts of the research trial the engagement is linked to. Discussions of the ethics of engagement and the role it can play are not often systematically linked to any practical examples of engagement being reported. Nevertheless, a small but significant body of social science and empirical ethics research on malaria trials explores engagement in greater detail, with some rich qualitative studies giving a clearer picture of some of the causal relationships and influential contextual factors involved.

### Clarification of analytical terms

In the literature reviewed, terms such as trust, participation and ownership are often used loosely in an everyday descriptive way, rather than in a consistently analytical way - what Sayer (2000) has called ‘poor abstraction’. Where possible in the review we attempt to find more specific terms and make their meaning clear. For example, we avoid making the notion of ‘trust’ central to our analysis, even though the language of trust occurs throughout the literature; finding instead that the notion of ‘working relationships’ gives a better account of the complex, precarious, ambivalent and negotiated relational dynamics involved in people coming to take part in research ( *cf* Leach & Fairhead, 2007). Conversely, we use the notion of ‘acceptance’ of research as an outcome of engagement in our analysis, but spell out that for us this usually means ambivalent rather than wholehearted acceptance ( *cf* Gooding *et al*., 2018b). The term ‘community’ is used in very different ways in the literature and has been subject to extended discussion in its own right in a number of academic disciplines (see Marsh *et al*., 2011 in relation to CE and Walkerdine and Studdert, n.d, for social theory). In our review, when we talk of ‘community members’, we refer to people living in the immediate localities affected by or potentially involved in malaria trials in LMICs. We refer to ‘local research stakeholders’ as those more immediately affected by or with an interest in research, such as participants, community leaders, and local health workers.

### Summary of programme theory

Our analysis suggests that CE involves a core logic of researchers developing working relationships with a range of local stakeholders affected by research, at different levels, and across differences (primarily of wealth, power, culture) and often in the context of suspicions and concerns about research. Such ‘working relationships’ are pragmatic and provisional relationships that develop through interactions around research initiatives, primarily between frontline research staff and local stakeholders, and through a combination of tangible research related benefits, and interactions that are experienced as relatively responsive and respectful.

These working relationships, which may commonly be experienced as ambivalent, contribute to greater acceptance and participation in research by local stakeholders, albeit with a range of different motivations and understandings of what participation means. Such working relationships are made up of four mutually reinforcing relational dynamics:

1. exchange of mutual benefits from research

2. contiguity and a sense of everyday presence and accessibility of research staff

3. a sense of influence or control over research for local research stakeholders

4. researcher responsiveness - and the degree stakeholders feel listened to and their concerns acknowledged

Shaping the nature of such working relationships are the policies and conduct of research groups and institutions on research and engagement, including commitment of senior research staff ensuring that engagement is adequately resourced, integrated into research management and initiated early, and the skills of frontline research staff who engage directly with local research stakeholders. Undermining the establishment of working relationships are aspects of what we call the ‘dominant health research paradigm context’, which includes: research having historical links with colonialism, coupled with recent history of more or less vertically imposed health interventions; research being externally funded, designed and controlled; contemporary differences of wealth, power and culture between research centres, and surrounding settings of poverty and under-resourced health systems. These influences tend to increase suspicion and lack of acceptance of research.

While the development of working relationships between researchers and local research stakeholders helps to get research done, it may also depend on suppressing formal recognition of inequalities and differences within research systems, and informal mitigation of them through research staff interactions with research participants, in order to maintain the flow of research related benefits. In this way, the very relationships facilitated by CE that help with research implementation, tend to reproduce some of the ethically problematic characteristics of the dominant health research paradigm. At the same time, a strand of our analysis highlights how a different dynamic of ‘collaborative partnerships’ around research, and greater routine access to health and health care in LMICs, may be pre-requisites for more ethical engagement in research.

## Main areas of analysis

**Figure 2.**
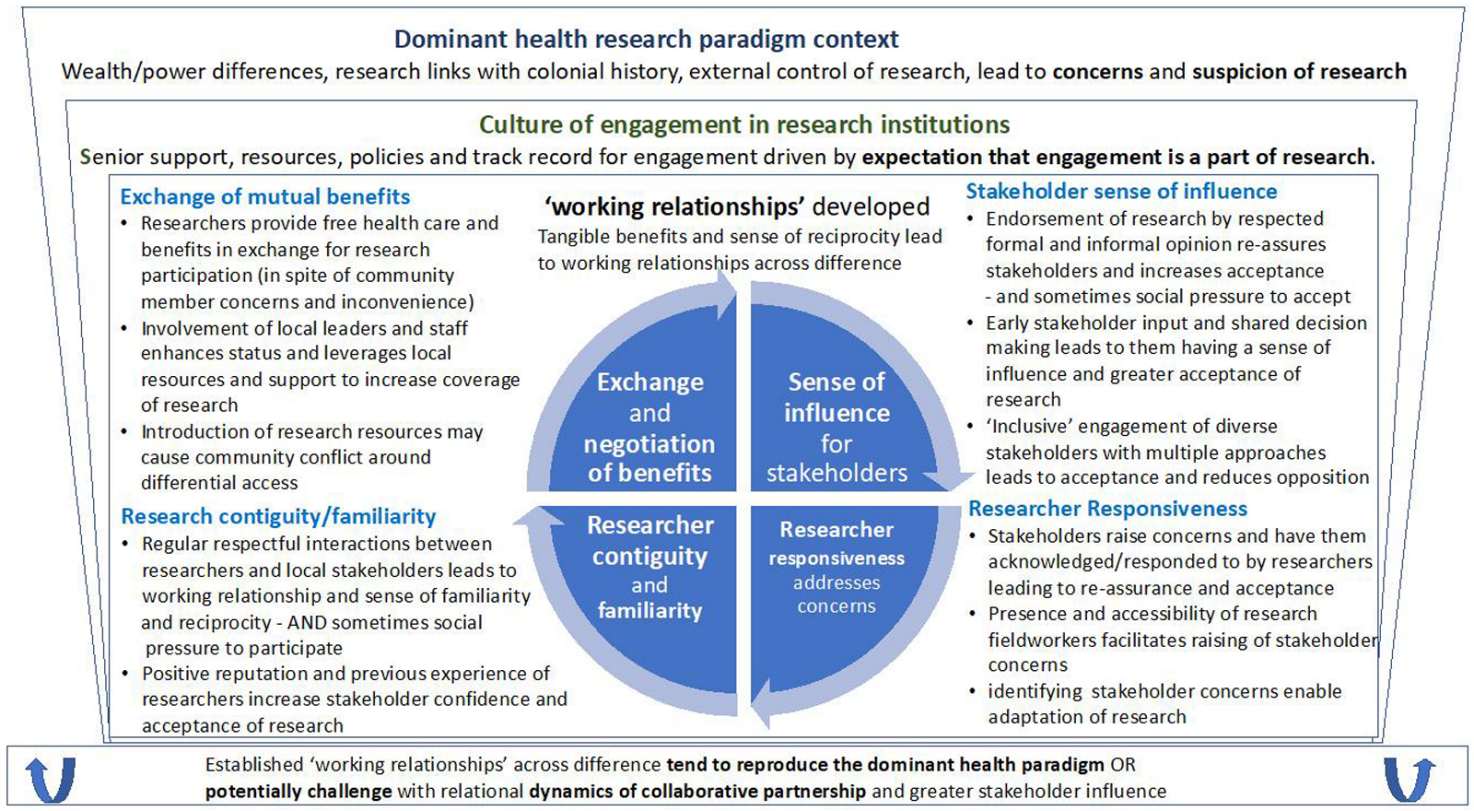
Programme theory summary – engagement developing ‘working relationships’ across difference.

Figure 2 below summarises how the different part of the analysis come together overall and Table 2 shows the 12 overarching CMOCs that combine together in our programme theory. Our analysis developed iteratively, from 60 initial detailed CMOCs, that were then organised into 25 clusters of CMOCs, and further grouped under 12 overarching CMOCs at a middle level of abstraction. Appendix 5 in *Extended data* provides a full table of all the CMOCs.

**Table 2.** Main overarching Context Mechanism Outcome Configurations in REAL analysis.

Analysis areas	Main Context Mechanism outcome configurations (CMOCs)
**Working relationships established**	
	CMOC1: When researchers develop working relationships with local research stakeholders (C) and provide tangible benefits (C) local research stakeholders may show increased acceptance of research (O) because they are re-assured by the sense of relationship and reciprocity with researchers (M)
**1. Exchange and** ** negotiation of benefits**	CMOC2: When researchers in resource poor settings provide free health care and other personal and community benefits, compensation and incentives (C) this may increase participation in research (O) when the benefits provided are seen by people as an acceptable exchange for their time, inconvenience, risk, blood,samples (M) or when research related benefits are seen as irresistible (M)
	CMOC3: Involving leaders and organisations based in study settings in research (C) and recruiting and training people from those communities as research staff (C) may reduce costs (O) and support research implementation (O) because it leverages local personnel, infrastructure and practical support for research (M)
**2. Researcher contiguity** ** and familiarity**	CMOC4: The everyday presence of community-based research staff (C) and repeated respectful interactions, both formal and informal, between community members and research staff (C) can lead to working relationships between researchers and community members (O) because people feel a sense of familiarity and rapport (M) and degree of reciprocity (mutual respect and understanding) (M)
	CMOC5: When participants are approached by research staff who they have a good previous experience of (C) or who have a good reputation and perceived competence (C), they are more likely to participate (O), because of increased confidence in those doing the research (M)
**3. Sense of influence or ** **control over research**	CMOC6: When research is endorsed by people, networks and/or organisations that community members have confidence in (C) they are more likely to accept or participate in the research (O) because they are re-assured by the endorsement (M) they feel leaders have some influence on researchers (M) or feel socially pressured (M)
	CMOC7: Where community members have early involvement (C) and share decision making in the design or conduct of research (C) they may contribute time and resources (O) and research may address locally relevant issues (O2) because they have a sense of influence over the process (M) and are motivated to identify challenges and solutions (M)
	CMOC8: Researchers working with a wide range of people in a community (C) and using multiple and complementary forms of representation (C) can lead to stakeholders being more likely to accept research (O) and share their views with researchers (O) since all groups feel they have been considered (M)
**4. Researcher** ** responsiveness**	CMOC9: Where local research stakeholders have regular opportunities to raise concerns and have them acknowledged and responded to (C) they better accept/tolerate research (O) because they feel re-assured by having their concerns taken seriously (M) and feel respect has been demonstrated (M)
	CMOC 10: When researchers identify community members’ concerns, beliefs and practices (e.g. using formative research, knowledge of locally embedded staff and ongoing CE) (C), research implementation is likely to be improved (O), because researchers can adapt the research to address local practical and social issues (M)
**5. Culture of engagement in research institutions**	
	CMOC11: Where senior researchers see engagement as important (C) and/or there is a culture of and infrastructure for engagement (C) researchers will more likely dedicate time and resources to engagement (O) because engagement is an expectation for those conducting research (M)
**6. Dominant health paradigm context**	
	CMOC12: Where there are differences of wealth, power and culture between research centres and local research stakeholders (C) and previous negative experiences of colonialism or outside agencies (C) people may be suspicious of research (O) because they perceive research as having an exploitative or hidden agenda (M)

In the following sections we outline each of the 12 overarching CMOC in turn, beginning with those focused on the core dynamic of developing ‘working relationships’ (CMOCs 1-10). These CMOCs overlap and interact with one another and are inevitably shaped by the final two overarching CMOCs (CMOCs 11-12) focusing on institutional and dominant health research contexts. For each overarching CMOC, we also reference ‘additional’ CMOCs that provide additional nuanced detail and are well supported by data, labelled with supplementary letters (e g CMOC1a) and which can be found in Appendix 5 in *Extended data*.

## Analysis - Developing ‘working relationships’ across differences

Our analysis suggests that it is a combination of the exchange of mutual benefits AND interactions that create a sense of personal reciprocity that lead to the development of ‘working relationships’ between research staff and local research stakeholders. In this way, working relationships are importantly underpinned by tangible exchanges.

CMOC1: When researchers develop working relationships with local research stakeholders (C) and provide tangible benefits (C) local research stakeholders may show increased acceptance of research (O) because they are re-assured by the sense of relationship and reciprocity with researchers (M)

Conversely, where short funding and research cycles limit the depth and duration of community and stakeholder interactions, it makes it difficult to establish working relationships, and this makes people less likely to accept research (CMOC1a, Appendix 5 in *Extended data*).

An additional strand of our analysis (CMOC1b) suggests that another contributor to this sense of relationship was a feeling of being cared for – created by the provision of relatively high-quality health care, treatment and support for research participants. An important context for this sense of being cared for, was the fact that by comparison, constrained resources and time in the public health care system limited the ability of staff to give time and attention to individual patients.

The following 4 sections look in more detail at several interacting dynamics that contributed to the sense of working relationships between research staff and other local research stakeholders.

### 1. Exchange and negotiation of benefits

In this section we consider a range of benefits that local research stakeholders saw as flowing from their participation in research, and the benefits researchers gained from stakeholder acceptance and participation in research

CMOC2: When researchers in resource poor settings provide free health care and other personal and community benefits, compensation and incentives (C) this may increase participation in research (O) when the benefits provided are seen as an acceptable exchange for participants time, inconvenience, risk, blood, samples (M) or when research related benefits are seen as irresistible (M)

It is striking from the literature how much local research stakeholders conceive of their participation in research as an exchange: in which they gain access to valued health care in the first instance, and a range of other material benefits as individuals and for the communities of which they are part; in exchange for their time, inconvenience, risk, and in many cases blood (which can be of significant concern – something we return to below). In contexts where health systems are weak and there is relative poverty, the access to health services accompanying research participation are a strong incentive for local stakeholders.

“ *What attracted us [was that] we knew our children will receive treatment for a whole year in every disease they suffer. If you have a problem and visit the people concerned, a call is made to the [PrincipIe Investigator] he brings a vehicle and [the sick person] is carried away [to hospital]. In fact it’s something we should be happy about because nobody can bring you a vehicle that easily.*

*(Mother 2, FGD 3)”* ( Marsh *et al*., 2011: 34)

Additional benefits accompanying research participation include reimbursement for transport costs, other material benefits and refreshments. Community level benefits linked to particular studies may include additional staffing and supplies, and some research centres may make longer term contributions to local health infrastructure and skills, and their provision of local employment may also be considered a community benefit.

Community members who are being asked to take part in research weigh up whether these benefits are an acceptable exchange for their participation, time and inconvenience. Sometimes the benefits are too great to resist, even in the face of misgivings or concerns. And sometimes the opportunity costs or risks of being involved in research are seen to outweigh the benefits. Benefits may also be subject to negotiation and inflation, depending on the particular context and opportunity costs, and local estimations of what makes for an acceptable exchange. Monetary incentives may sometimes be seen as required for adequate compensation, and at other times as signalling potentially greater risks for participants, particularly in the absence of established relationships of some sort, and where money may be seen as a substitute for a relationship.

‘Acceptance’ of research is often ambivalent and with reservations, more ‘toleration’ ( Kolopack *et al*., 2015) rather than wholehearted acceptance without qualification (cf Gooding *et al*., 2018b). Prospective participants usually have a range of concerns and sometimes serious misgivings about participation – commonly around research procedures involving drawing of blood - but these are outweighed by the interest in access to health care. In this way, research related benefits are irresistible for some, reducing their ability to make a choice without undue influence (CMOC2d).

Additional strands of analysis suggest that stakeholders’ overriding interest in accessing health related benefits mean that clear understandings of the research they are involved in are not a priority or are ‘crowded out’ by this primary interest (CMOC2c). There are a few clear examples of a ‘therapeutic misconception’, where the primary aim of research is misunderstood as treatment. However, more commonly, people are simply motivated to participate in research to access the very real health benefits that come with participation, and their understanding of the research is secondary.

Local employment is commonly seen as a benefit in itself by research communities (CMOC2b), and local employment can lead to community recognition and status for local fieldworkers and research staff, especially in contexts where community service roles are valued (CMOC3.2a). 

***Research resources fuelling community or household tension.*** At the same time, who gets access to local employment can be contentious, consistent with another strand of analysis highlighting how the introduction of research related resources and benefits can cause household and community conflict (CMOC3.3). Taking part in a study can be seen as ‘selling out’ to outsiders or aligning with research institutions in order to gain access to benefits, which can at the same time become a source of jealousy. In a number of studies involving children, there was a tendency for those with caring responsibilities to favour participation for the access it gave to health care, while other household members or relatives might be less in favour and stress concerns over involvement.

Introduction of research resources may put some local people in a position where they ‘broker’ access to these resources on behalf of others. The notion of brokerage ( Tilly, 2005) is useful for the way it draws attention to the dynamics of intermediary roles in exchanges. In our analysis, brokerage features in how local fieldworkers or authorities balance supporting research goals (e.g coverage/recruitment), and satisfy local interest (e.g in accessing health care and other benefits), while simultaneously enhancing their own status. In a few instances, resources were reportedly brokered by intermediaries for their own agenda and influence, leading to research being associated with particular groups or factions and to uneven research implementation (CMOC3.3a).

From the available data it is not clear under which circumstances research resources were more likely to become a source of conflict. Some papers suggest that where people do not expect their relationship with a research programme to be ongoing they prioritise immediate gain over the potential longer-term benefits of a relationship with the research programme. Njue *et al*. (2014) suggest that in settings where community life is imbued with a spirit of voluntarism and cooperation, provision of a variety of material benefits may seem to contradict this ethos and so create a divide between those who do and do not receive them in exchange for participation. In such contexts, maximising benefits that apply to the whole community, such as strengthening of existing health care infrastructure may be a way to mitigate these divisions ( Molyneux *et al*., 2012a; Njue *et al*., 2014).

CMOC3: Involving leaders and organisations based in study settings in research (C) and recruiting and training people from those communities as research staff (C) may reduce costs (O) and support research implementation (O) because it leverages local personnel, infrastructure and practical support for research (M)

Our analysis also highlights how research related exchanges support research. Recruitment and employment of local people is a way to reduce costs and increase coverage, and involving local leaders, important for negotiating ‘permissions’ for research and leveraging local participation and resources to improve implementation (such as meeting space). It also more or less explicitly aimed to leave a legacy of greater local skills and infrastructure for health as a benefit of research (CMOC3.1a)

### 2. Researcher contiguity and familiarity

CMOC4: The everyday presence of community-based research staff (C) and repeated respectful interactions, both formal and informal, between community members and research staff (C) can lead to working relationships between researchers and community members (O) because people feel a sense of familiarity and rapport (M) and degree of reciprocity (mutual respect and understanding) (M)

Repeated respectful interactions between research staff and local research stakeholders can contribute to a sense of relationship and familiarity. Such interactions may be part of formal engagement activities and events, but more often it is the everyday presence and informal interactions of locally recruited fieldworkers – their ‘contiguity’ ( Maxwell, 2012) - that play an important role in developing relationships with local research stakeholders. Such interactions include the sharing of food and attendance at social events. This sense of reciprocity is sometimes illustrated by relations with research fieldworkers being expressed through family and kinship terms.

“ *Over time… there was a shift in interactions from that of formal professional to one infused with informality and relatedness. Familial titles such as daughter, son, grandchild, were used to describe the types of relationship that were evolving between FWs [fieldworkers] and participants in the negotiation of study procedures. Requests by participants for benefits and gifts beyond those officially provided by the study, such as for food items, cell phone airtime, and baby clothes, became increasingly common. FWs were sometimes also consulted on non-study related issues such as land ownership, planned community development projects and mentoring of young people.*” ( Kamuya *et al*., 2013)

Such relationships may be influenced by the degree of ‘embeddedness’ of local research staff - which may vary from living and working in the communities being researched, to living in a central town and travelling out to work in study communities nearby. Even where a fieldworker lives in a particular community, social networks may be complex and multiple, and the actions and perceived affiliations of an individual fieldworker may have implications for the wider acceptability of research they are associated with ( Molyneux *et al*., 2010: 25).

Realist social theory highlights the important role of regular interactions – contiguity – in the development of relationships and a sense of community, in contrast to the often-assumed role of a perceived similarity in social characteristics or ‘homophily’ ( Maxwell, 2012:54; Sayer, 2000). They argue that concrete interactions, and the real exchanges and negotiations, shared presence and time, are a strong basis for developing relationships, compared to the ‘virtual’ relationships of similarity. In the literature reviewed, fieldworkers being recruited locally was often suggested to increase acceptance of research without a clear rationale, and potentially an assumption that homophily leads to mutual understanding and some form of ‘trust’. Our data support the idea that it was frequent interactions over time that contributed to an emerging sense of relationship, rather than just a sense of similarity. As the example above illustrates, the complexity of local networks and allegiances, challenges any assumption that being ‘local’, necessarily leads to an immediate rapport between fieldworkers and community members.

At the same time, the development of expectations of reciprocity that come with relationships can also sometimes contribute to social pressure to participate (CMOC4b). Potential research participants may also appear to agree to participation in order not to disappoint the fieldworker, while actually participating only partially or not at all in research as intended – described as ‘silent refusals’ ( Kamuya *et al*., 2015).

Still another strand of analysis suggests that dedicated engagement staff who are a reliable and consistent point of contact for research stakeholders, can also make research feel more accessible and help to build relationships (CMOC4d).

CMOC5: When participants are approached by research staff who they have a good previous experience of (C) or who have a good reputation and perceived competence (C), they are more likely to participate (O) because of increased confidence in those doing the research (M)

Previous positive experiences of researchers or research programmes and institutions over time, and their perceived good reputation and competence encourages local stakeholder confidence in research. 

### 3. Sense of influence over research for frontline research stakeholders

In the literature reviewed our analysis showed that local research stakeholders sometimes had a sense of influence over research, but rarely any formal or explicit control or decision-making in research processes.

CMOC6: When research is endorsed by people, networks and/or organisations that community members have confidence in (C) they are more likely to accept or participate in the research (O) because they are re-assured by the endorsement (M) or feel that leaders have some influence on researchers (M) or feel socially pressured (M)

Where research is endorsed by a range of local leaders, influential household members and neighbours who people have confidence in, this re-assures community members and encourages them to accept research. Rather than explicit control over research, this acceptance by local opinion amounted to a sense of research being less of an unknown quantity and in a sense more under local influence.

*“[It was] good because he passed through the government; we saw him first with the chief. That made us feel peaceful because he was with the chief, a village elder and our hearts were clean because we know if any bad thing befalls us, we’ll first get hold of the chief or the village elder to solve that problem (K1, P3/7, page 19).” ( Gikonyo *et al.*, 2008: 712)*

Our analysis highlights that these same sources of influential local opinion are part of multiple and complex relational influences on individual decision-making in relatively ‘communal’ rural communities. These include patterns of household and family authority and responsibility, often based on gender and age, which vary across different geographical and cultural setting. Opinions and decisions are sometimes influenced by other people as figures of authority, but also through an expected process of iterative discussion and dialogue at the community and neighborhood level to inform decisions not only based on hierarchal influence (CMOC6b). Sometimes the endorsement of local opinion could also contribute to social pressure to accept or participate (CMOC6c). Our analysis underlines the importance of understanding local norms and practices of decision making and highlights challenges for more simple, one-off informed consent procedures.

CMOC7: Where community members have early involvement (C) and share decision making in the design or conduct of research (C) they are more likely to contribute time and resources (O) and research may address locally relevant issues (O2) because they have a sense of influence over the process (M) and are motivated to identify challenges and solutions (M)

While a considerable volume of discussion in the literature reviewed raised the question of to what degree engagement could provide research stakeholders with decision making power and control in the research process, there were very few empirical accounts of processes of decision-making in or explicit accountability of research. A small proportion of the data in the literature searched came from primary health care interventions where the process of the intervention encouraged local control over priority setting and involvement in problem solving in relation to local health challenges and this motivated sustained participation. This data provided the most concrete examples of community members having some control, although this was embedded in the intervention being studied, rather than in the research process. In addition, a couple of case studies of research with a more participatory design showed local research stakeholders having decision-making power in the research process itself ( Musesengwa *et al*., 2018). More commonly, local research stakeholders could have influence in implementation and research procedures (rather than research priorities or design). Such input could include reviewing of information and communication materials, procedures for informed consent, and provision of study related benefits for participants.

Where the literature reviewed looks at the role of community advisory boards (CABs) the data are largely descriptive and, with one exception, do not look systematically at issues of control or accountability. Members of CABs are often described as a ‘bridge’ between local communities and researchers; however there is little detail on how CAB members play a ‘representative’ function, and in a few examples, it was suggested that CABs, especially where members are selected through existing organisations and groups, could disproportionately serve the interests of those particular groups ( Abimbola, 2020; Kamuya *et al*., 2013). Another strand of analysis suggests that where engagement platforms are funded and led by researchers, this may compromise their independence, particularly where there is no explicit expectation or processes for research accountability (CMOC8.1).

CMOC8: Researchers working with all sides in a community (C) and using multiple and complementary forms of representation (C) means stakeholders are more likely to accept research (O) and share their views with researchers (O) since all groups feel they have been considered (M)

Another related strand of findings highlights the importance of research not being perceived as affiliated with one particular ethnic, political or religious group. Where it is clear that all sides and factions (in the case of politically or religiously divided communities) have been engaged and included in research engagement, through a range of different approaches appropriate to those groups, research may more likely be widely accepted. Such an ‘inclusive’ approach is less likely to provoke opposition of particular groups who feel left out. An inclusive approach to engagement is also practically valuable for researchers, who are more likely to be made aware of a range factors to inform adaptation of research implementation (CMOC8e).

### 4. Researcher responsiveness

CMOC9: Where local research stakeholders have regular opportunities to raise concerns and have them acknowledged and responded to (C) they better accept/tolerate research (O) because they feel re-assured by having their concerns taken seriously (M) and feel respect has been demonstrated (M)

Research stakeholders may be suspicious of research for a range of reasons rooted in the dominant health paradigm context which we return to below, including negative experiences of colonial history, more recent vertical health interventions, or contemporary differences in wealth, culture and power between research centres and local research stakeholders. In addition, our data highlighted that blood tests/samples in particular, and unfamiliar research procedures, may be a cause for concern or experienced as relatively onerous. When such concerns remain unaddressed by researchers this can undermine acceptance of research and also contribute to the spread of rumours. Our analysis highlights that when local research stakeholders can raise their concerns and have them acknowledged, addressed or responded to, they will more likely feel re-assured and respected, ultimately increasing acceptance of research.

*“the most popular mechanism was face-to-face presentations, like those used in the focus groups, which also provided time for those present to ask questions of the scientists, reflect on their answers and hear other community members’ views...Recurring questions or concerns were fed back to the scientific team, who undertook new experiments and prepared responses to questions during community presentations that were adapted for use in communication materials” ( McNaughton, 2012: 5-6)*

Researcher responsiveness may be demonstrated during formal engagement activities or ongoing forums such as CABs, where stakeholders are able to raise concerns and ask questions in an open, respectful atmosphere, and have these discussed, acknowledged and responded to. Equally, responsiveness may be more informal, facilitated by locally recruited research fieldworkers whose presence and accessibility for community members may make it easier to raise and respond to concerns.

Where researchers respond to concerns by providing explanations and information, our analysis suggests that this may be as important for the way it can re-assure people by demonstrating they are being taken seriously and showing respect, as it is for improving stakeholder understandings of research. Provision of accurate information and the need to tailor it to local context and communication channels were often discussed in the literature reviewed, but, perhaps surprisingly, were rarely explicitly linked to engagement outcomes. Conversely, overly focusing on provision of information may actually confuse and overwhelm stakeholders, who tend to interpret research through their own frames of reference and local beliefs and understandings (CMOC9f-g).

CMOC10: When researchers identify community members concerns, beliefs and practices (e.g. using formative research, knowledge of local staff and ongoing CE) (C), research implementation is likely to be improved (O), because they can adapt the research to address local practical and social issues (M)

Our analysis suggests that formative research and drawing on the knowledge of local staff and fieldworkers can help to adapt research implementation, by identifying salient cultural beliefs, social practices and stakeholder groups that need to be accommodated by the research process. This process of being made aware of important local issues is also enhanced where communities and stakeholders have regular opportunities to raise concerns and issues through the formal and informal processes highlighted above.

### 5. Culture of engagement in research institutions: leadership, commitment to and resources for community and stakeholder engagement

CMOC11: Where senior researchers see engagement as important (C) and/or there is a culture of and infrastructure for engagement (history and institutional policy and roles dedicated to) (C) research will more likely dedicate time and resources to engagement (O) because engagement is an expectation for those involved in conducting research (M)

Our analysis suggests that where senior researchers or the wider research institution has a commitment to engagement, this increases the likelihood that it is resourced, and capacity developed to design and implement it. Facilitative leadership of senior staff is key to this commitment, as are institutional policies and roles and a practical track record of engagement, which may add up to a culture of engagement and an expectation that it is part of research. Commitment to engagement is also manifest through: dedicated roles for engagement that act as a consistent point of contact for stakeholders; drawing on technical inputs on engagement from social scientists and other experts; and investment in processes of reflection and evaluation that inform management of engagement and research (CMOC11a-c). Such commitment is facilitated where funders make resources available for engagement, and have an explicit expectation that research will be accompanied by engagement (CMOC11.3). Given the complex intermediary role of research fieldworkers, supportive supervision and participatory training based on experiential learning for research fieldworkers, is another important part of engagement infrastructure (CMOC11.2).

Where researchers insist that local research stakeholder input informs the design and delivery of research, this tends to inform the choice of engagement methods used such that they are more responsive to those inputs (CMOC11.1). Conversely, where engagement is poorly understood by researchers this may increase the likelihood of it being under-costed, and where researchers do not see engagement as important, they are less likely to do it (CMOC11d-e).

### 6. Dominant health paradigm context

CMOC12: Where there are differences of wealth, power and culture between research centres and local research stakeholders (C) and previous negative experiences of colonialism or outside agencies (C) people may be suspicious of research (O) because they fear research has an exploitative or hidden agenda (M)

As noted above, CE with health research takes place in LMICs against a backdrop of what we call a ‘dominant health research paradigm context’ characterized by a number of features. It is a context where the communities where research is taking place are often relatively poor and with under-resourced health systems, and where research centres are among the more wealthy and powerful institutions, often linked into international networks. In addition, there may be previous negative experiences of colonialism and external intervention, including recent experiences of top-down health interventions led by - or with a strong presence of - outside agencies. Such differences of wealth and power can lead to prevailing suspicion of research for community members and local research stakeholders.

Also contributing to this suspicion of research are the ways in which research is experienced as externally funded, designed and controlled - which may lead local research stakeholders to read contemporary research relations through previous negative experiences. Three additional CMOCs highlight: research agendas being defined by a network of external actors; inflexible research protocols - often tied to biomedical trial designs and to external and national ethical approvals limiting the scope for local input; and researchers from the global south having less influence over research design compared to researchers from the global north with greater capital and status (CMOC12.1-12.3)

Two additional features of the dominant paradigm of health research are: 1) the tendency for governments and public authorities engaged in research faced with reduced public confidence being keen to support engagement to bolster their perceived legitimacy (CMOC12.4); and 2) emphasis on individual autonomy/choice and the researcher-participant interaction in research ethics, which tends to obscure the structural constraints on personal decisions and overlook some of the differences of wealth and power in the research encounter (CMOC12.5).

## Discussion

Below, we situate some of the findings from our review of CE in malaria research within wider debates around CE with health research in LMICs. We highlight how the conceptual resources drawn on in the review inform the analysis and draw out some of the implications of our programme theory for CE with health research more broadly. We highlight how CE is a meeting point for diverse stakeholders across differences of wealth, power and culture. The relational core of engagement makes engagement a messy, negotiated process which can blur the boundaries between formal and informal interactions, and put research fieldworkers in a complex intermediary position. At the same time, the ‘terms of engagement’ in CE mean that researchers largely retain control over the research process. Relatively wealthy research institutions offer access to locally desired health care in settings where health systems are under-resourced, yet this and other influences on people’s decision-making tends to be obscured by research ethics focusing on individual informed consent. Research stakeholders may overlook the inequalities between them to preserve the precarious working relationships that maintain the flow of research related ‘benefits’. In this way, such working relationships tend to reproduce characteristics of the dominant health paradigm context. A lesser strand of our analysis hints at an alternative dynamic of ‘collaborative partnership’ with community members and local research stakeholders, where there is open recognition of and work to challenge the dominant health paradigm context. Such collaborative partnerships have been argued by many to be central to realising the social value of research ( Emanuel *et al*., 2004).

### Different understandings and purposes of engagement

Community and stakeholder engagement with health research in LMIC settings is perhaps best understood as a meeting point of different stakeholders around a research intervention ( Long, 2001; Molyneux & Geissler, 2008). In engagement interactions around this common focus of research, different groups draw on their own social networks, conventions and resources ( Crossley, 2011). Our analysis highlights how the meanings and purposes of engagement and the relationships and resources exchanged may be different for different research stakeholders. Oversimplifying, researchers may have an interest in getting good quality research done in a way that is as ethical and as quick as possible, while the primary interest of local research stakeholders is access to health care, which means that they participate in research despite sometimes considerable misgivings.

Anthropological accounts in African contexts highlight how local beliefs about researchers stealing or selling participants’ blood taken during research procedures (which come up frequently in the literature), relate to concrete concerns and real relations between wealthy research institutions and local people, expressed through local idioms of blood and exchange ( Fairhead *et al.*, 2006; Geissler & Pool, 2006; Kingori *et al.*, 2010). At the same time, in settings where local authorities or the national state are experienced as operating through patronage, research related resources can be drawn into such patronage relationships ( Leach & Fairhead, 2007) something that is also evident in our review literature from South East Asia.

While there is an emphasis in the literature on researchers addressing misunderstandings of biomedical concepts and research through provision of accurate information, we find little clear evidence that accurate understanding of research studies plays a main role in decisions of whether or not to participate. Rather, participation seems to depend more on the re-assurance provided by ongoing relationships and interactions that are responsive and respectful (where provision of information may be an important part of showing respect). The perceived good reputation of researchers is also important as we have shown. Our analysis suggests efforts to understand local beliefs and practices relevant to health and research in depth continue to be needed, both to demonstrate respect, and to better respond to local concerns in research procedures where possible ( Leach & Fairhead, 2007; Nuffield Council on Bioethics, 2020; Vanderslott *et al*., 2021).

### Relational emphasis in engagement

Our analysis supports a strand of engagement scholarship that sees stakeholder relationships as a crucial practical foundation for research initiatives ( Geissler & Molyneux, 2011; Gikonyo *et al*., 2008; King *et al*., 2014). In the context of uncertainties and concerns, and negotiations over the terms of people’s engagement in research, relationships with local research staff are an important source of re-assurance ( Angwenyi *et al*., 2014). This strand of scholarship sees CE in practice as a messy, negotiated process developed through formal and informal interactions over time, rather than a discreet technical intervention, guaranteed by particular activities and procedures. Effective engagement may involve more than formal activities, and even in the latter case, it is often the quality of interactions – open questioning, listening and respectful discussion despite existing power relations – that lead to an emerging sense relationship, rather than the activities themselves.

Our analysis also supports claims that research fieldworkers play a crucial and creative role in ‘doing ethics’ on the ground in the concrete settings of research, and that this role is often under-recognised and under-supported ( Kamuya *et al*., 2013; Kamuya *et al*., 2015; Kingori, 2013; Molyneux *et al*., 2010). Some scholars argue the ethical challenges of engagement are effectively ‘outsourced’ to the interpersonal negotiations between fieldworkers and local research stakeholders ( Kingori, 2015). Given the ambiguous and challenging role taken on by fieldworkers, there is a need for greater supervision and institutional support, as well as professionalization and development of related career pathways ( Kombe *et al*., 2019; Molyneux *et al*., 2013). Such support for engagement staff appears to be most developed where there is a commitment to engagement at an institutional level over time ( Kamuya *et al*., 2013; Kombe *et al*., 2019; Lairumbi *et al*., 2012; Njue *et al*., 2014). Commitment to engagement, in turn, relies on the facilitative leadership on the part of senior researchers and research institution directors, ensuring engagement is a priority and resourced adequately ( Aggett, 2018; Boulanger *et al*., 2020; De Weger *et al*., 2018; Evans *et al*., 2014; Kolopack *et al*., 2015). The importance of such leadership was most clearly documented in reviews of engagement in settings in the global North that we drew on in developing our initial programme theory, but this is complemented by some LMIC data and direct knowledge from various members of the team in LMICs.

The complex relational character of engagement may also be one of the reasons for the underdeveloped state of evaluation of CE ( Aggett *et al*., 2012; Gooding, 2018b; Lavery, 2018; MacQueen *et al*., 2015; Newman, 2006). Our analysis suggests that where CE is informed by social science and other technical expertise, it may better attend to the complex relational dynamics involved. The lack of conceptual consistency identified in our review however, highlights the need for greater explicit attention to the assumptions and theory of change underpinning engagement in practice. Our review demonstrates the value of attending to the causal dynamics of CE using a realist logic of analysis. There may also be useful lessons to draw from the international development literature for strengthening the evaluation of CE ( Vincent, 2012).

***Terms of engagement.*** If our analysis highlights the importance of relational dynamics in CE it also shows that the ‘terms of engagement’ under which people may accept or participate in research are largely set by researchers. International development scholars have demonstrated how the terms on which people are engaged in the ‘spaces of participation’ constituted by development encounters, are often set by more powerful actors, and limit and shape the possibilities for equitable negotiations for the less powerful in such spaces ( Cornwall 2008; Gaventa & Cornwall, 2006). In the case of CE, researchers bring considerable material resources, in terms of the access to health care and other research related benefits and compensations, and largely control the funding, design and delivery of research and associated engagement mechanisms and ethical procedures.

***Political economy and history.*** The terms of engagement around health research are also influenced by the history and political economy of health research in LMICs, as well as contemporary experiences of government disregard. The prevailing suspicion of health research identified in our review chimes with historical accounts of the intimate relationship between health research and colonial administration, as well as experience of vertical and sometimes coercive health campaigns during the colonial and early post-colonial period ( Packard, 2000; Tuhiwai-Smith, 2021). Literature exploring the political economy of CE highlights how health research is often carried out by wealthy research centres in settings of relative poverty and how the immediate researcher – stakeholder interactions of engagement are nested in a set of wider social-economic and power relations which limit stakeholder influence and control ( Fairhead *et al.*, 2006; Reynolds & Sariola, 2018).

***External control of research.*** Much health research is funded and managed by international research partnerships, which can constrain national governments’ interest and ability to set and follow local research agendas ( Birn, 2014; Clinton & Sridhar, 2017; Lairumbi *et al*., 2008). These international partnerships, typically underpinned by funding from the North, are often led by Northern researchers; power differences, including institutional hierarchies within research centres, can reduce Southern researchers’ decision-making power, and their access to and control over research infrastructure and facilities ( Participants at CE workshop, 2013; Lavery & Ijsselmuiden, 2018; Parker & Kingori, 2016). Such power differences and hierarchies are also reproduced within regions, countries and institutions, with those with external links and networks in relatively powerful positions locally.

An additional aspect of health research that may add to local stakeholders’ sense of external control is the character of the biomedical trials that tend to dominate internationally funded health research in LMICs, and which are the predominant focus of our review. Such trials tend to have inflexible research protocols dictated by the need for controlled comparisons amenable to particular statistical tests. In this context, community and stakeholder input may be limited to tailoring of research implementation, something which has been explicitly highlighted in reviews of engagement undertaken in the North ( Evans *et al*., 2014), but which may also be characteristic of trials undertaken in the South

In the literature reviewed, concerns about levels of stakeholder control in research are a common theme reflected in discussions of typologies such as Arnstein’s ladder of participation ( Arnstein, 1969) or more recent ethical frameworks that advocate meaningful stakeholder involvement throughout the research process ( CIOMS, 2016). However, these frameworks are rarely linked to particular instances of engagement or assessment of engagement outcomes. There are also few detailed discussions of representation or accountability, as compared to processes of consultation, gaining endorsement, or involvement in research procedures, with a few exceptions ( Kamuya *et al*., 2013; Pratt *et al*., 2015 - examining the Community Advisory Board established by Cheah *et al*., 2010, and Schairer *et al*., 2019). These examples highlight the challenge of finding processes for representation and accountability in research that are both procedurally robust and acceptable to the range of stakeholders being engaged by researchers. At the same time, they highlight how, in the context of large malaria trials, few attempts to address this challenge have been documented in the literature.

***Individualist research ethics.*** Another aspect of the dominant ‘terms of engagement’ is the focus of bioethics on individual autonomy and informed consent, which tends to ignore the wider influences on people’s agency and decision making. Recent critiques argue that in practice, participants often experience undue inducement, and are offered an ‘empty’ choice, or ‘pretence’ of choice ( Hoeyer & Hogle, 2014: 35; Kingori, 2015: 774). Our analysis suggests that research participation in such contexts is often experienced as an exchange for access to health care that is otherwise limited. This is consistent with a strand of scholarship highlighting how CE with health research in LMICs may be influenced by wider inequalities outside the immediate research-based relationships ( Molyneux & Geissler, 2008) and amount to what has been called structural coercion ( Kingori, 2015; Nyirenda *et al*., 2020).

Debates about a ‘fair offer’ for research participation ( Nuffield Council on Bioethics, 2015) have highlighted the importance of managing study related risks, which may include both undue inducement, and exploitation of participants if they are not adequately compensated ( Emanuel *et al*., 2005; Njue *et al*., 2014). Practical policy recommendations flowing from this debate include making direct benefits primarily medical, while at the same time aiming to maximise the collateral benefits to whole communities, among others ( Molyneux *et al*., 2012b). In addition, researchers and ethicists have sought to broaden the focus of research ethics beyond individuals and immediate research relationships, to consider the meso level of supporting health facilities, and macro level of health systems and social, political and economic constraints ( Hyder *et al*., 2012; Lairumbi *et al*., 2012; Lavery *et al*., 2010; Molyneux *et al*., 2012b; Njue *et al*., 2014). Recent debates on ethics in public health and health systems research go further still to suggest that research ethics should be grounded in ethics of social justice ( Benatar & Singer, 2010; London, 2005; Pratt *et al*., 2020b; Powers & Faden, 2006) or solidarity ( Pratt *et al*., 2020b).

In the field of CE, concerns to move beyond the individualistic focus of bioethics have informed changes to international bioethics guidelines which increasingly suggest some form of community consent or authorisation ( CIOMS, 2016; Nuffield Council, 2020). Engagement scholars have highlighted the importance of processes of multi-stakeholder deliberation over time as a way of enhancing consent process, while emphasising the difference between formal processes of representation and informal forms of community authorization. The latter may be neither democratic or transparent, and remain under-theorised ( Lavery *et al*., 2010; Marsh *et al*., 2011; Molyneux *et al*., 2012a). There have also been successive attempts to strengthen the procedural standards for meaningful input and control for research stakeholders, as reflected in iterations of Good Participatory Practice Guidelines ( UNAIDS, 2011), and more recently the UNICEF minimum standards for community engagement ( UNICEF, 2020) and work that seeks to strengthen community input into research priority setting ( Pratt, 2019).

***Constrained agency.*** Insights from engagement literature suggest that research ethics need to address the macro structural issues that place constraints on people’s decision-making, and look beyond the procedural choices available within the engagement encounter. Feminist scholarship on politics and constrained agency has highlighted the way people’s choices and agency may be shaped by the social conditions in which they live, affecting how they engage with formal procedures of representation. This work suggests that ideal procedural frameworks may appear neutral and universal, while side-stepping questions of how people’s motivations and priorities are shaped by structural inequalities ( McNay, 2007; McNay, 2014) and the way their agency may be expressed outside formal settings ( Campbell & Mannell, 2016).

Some scholars see the turn to engagement on the part of formal institutions as a response to the legitimacy crisis experienced by a variety of public authorities and the increasing permeation of scientific research by the private sector ( Blume, 2017; Rose & Rose, 2012). Engagement in practice may amount to a contradictory mix of real concessions to stakeholder interests, and the co-opting of stakeholders into the agendas of powerful institutions. Such an analysis echoes broader scholarship of contemporary power relations that draws on the Foucauldian notion of governmentality which suggests that initiatives promoting ‘community participation’, despite appearances, may be a technocratic means of governing ‘through [apparent] freedom’ ( Cooke & Kothari, 2001; Rose, 1999; Vincent, 2002). Discussions of the politics of engagement in health research in LMICs point to risks that CE may co-opt people into research agendas they have no control over, and argue for greater critical analysis to identify characteristics of engagement that have the potential to make it more transformative, inclusive and meaningful ( Reynolds & Sariola, 2018)

***Accommodation of inequalities.*** Our analysis suggests that the core dynamic of developing working relationships across difference and the ‘terms of engagement’ characteristic of CE, tend to maintain the dominant health research paradigm. Geissler (2013) has evocatively described how research stakeholders may actively overlook the inequalities between them to preserve the precarious working relationships that have been established. This process of ‘un-knowing’ may be driven by a shared concern to maintain the flow of ‘benefits’ for different stakeholders through the research encounter, and preserve an appearance of relatively equitable and respectful relationships. Relational sociology similarly highlights how initially instrumental or conflictual interactions may evolve into established relationships over time if they are perceived to be mutually beneficial in some way ( Crossley, 2011). In our analysis the very different interests, degree of control and understandings of research stakeholders are accommodated through engagement practices, and the relationships that develop with local research staff in particular.

Realist understandings of social change suggest that such inconsistencies of belief and interest may sometimes be accommodated and sometimes challenged, depending on the balance of power between different groups and the available cultural and structural resources. Further, wider social change can happen when small changes in either cultural meanings, group dynamics and relationships, and institutional and structural arrangements reinforce each other to produce a critical mass of inter-related changes ( Archer, 1995). A strand of our analysis of CE highlights how an ethos of respect for stakeholders and commitment to shared decision-making can set in train interactions and relationships that become a new context for further collaboration ( Jagosh *et al*., 2012; Musesengwa & Chimabari, 2017) and a potentially different dynamic. A consistent approach to shared decision-making across research initiatives and the agencies and partners involved in research, can create an ‘equity context’ that may be key to effective engagement ( Harris *et al*., 2015).

As noted above, our focus on biomedical trials with fixed research protocols made it less likely that we would access examples of stakeholders having greater decision-making in research. In participatory action research ( Black *et al*., (n.d.); Gilson *et al*., 2021; Loewenson *et al*., 2014) and community based participatory research ( Minkler & Wallerstien, 2008) engagement is an integral part of the research process, so community members have more control over research and knowledge production. Extending our review to consider literature in these areas and the substantial body of knowledge on engagement, empowerment and participation in global health initiatives more generally ( Nelson, 2019) could provide more data on the dynamics and limits of stakeholder decision making in research and how these link to wider social and political processes.

***Limitations of the analysis.*** The volume of literature retrieved meant that we were unable to complement our searches on large malaria trials with systematic literature searches of other research paradigms that embed stakeholder input, which were highlighted as potentially useful in our protocol. For the same reason we were unable to more systematically consider literature that described failures, challenges and problems with CE, in spite of awareness within our team of relevant material. There are also areas where lack of data limited our analysis, including what makes researchers value engagement and commit to it in practice; the role of community advisory boards; the empirical record of good participatory practice guidance in research studies; the impact of research centre policies on engagement; the challenges of addressing stigma. In the case of work on community advisory boards, knowledge of the team suggests there is literature beyond malaria trials that we were unable to access as part of the current review.

The literature reviewed was predominantly from Africa. It is striking how little literature there was from the Indian subcontinent, given the considerable CE work undertaken there. This may be because CE is seen as more operational and not necessarily seen as worthy of being written up as research in itself, independently from the trials that are usually the main focus of research papers.

Drawing on citations from existing literature to identify additional papers to strengthen our analysis tended to predominantly access papers from KEMRI-Wellcome, the Ethox Centre and Lavery and colleagues – all teams that have published a considerable proportion of the existing CE literature. In addition to the volume of literature produced by these teams, it is also a case that some of the programmes with which they work are distinctive for their relatively long-term commitment to engagement and engagement scholarship over time. In this way, our analysis arguably rests on data generated about a selection of places studied in more detail, and would benefit from being further tested in other settings.

Nevertheless, our review synthesized a large volume of literature based on systematic searching and a systematic transparent review process which enabled us to identify important key relational mechanisms underpinning engagement. Existing knowledge of social theory in the team, which was drawn on to develop and refine our programme theory, is both a strength and a potential bias. An example is how our analysis was influenced by awareness of more or less un-acknowledged individualistic, psychological and biomedical bias of the literature reviewed. Conversely, the team brought an understanding of social theory and of key relational dynamics in social interactions which appears to have been neglected in the engagement field to date. Input from our team of scholar practitioner ‘content experts’ was useful for developing our initial programme theory, for reviewing the analysis at various points, including at a ‘validation workshop’ towards the end of the review to sense-check the findings and identifying areas of the analysis that need further development.

## Recommendations

Our review is at turns both wide and deep: balancing a deeper understanding of particular aspects of engagement, with a wider appreciation of aspects of context affecting the dynamics of engagement. In this way the review provides a higher resolution picture of the overall landscape of complexity of CE than has been available to date. Inevitably, the latter ‘wide’ dimension can only be broad brush, beginning to outline important distinctions and connections, and drawing attention to areas that provide a foundation for improvements in policy and practice as well further research in a number of areas. Where the data enabled us to go into more detail we point towards recommendations in a number of areas of engagement practice. We lay out some of these recommendations in Table 3, loosely grouped around different audiences, while a number of them are relevant to more than one audience.

**Table 3.** Researchers and engagement practitioners.

Researchers and engagement practitioners	
**Clarity around engagement purposes and outcomes**: our findings suggest a need for researchers and engagement practitioners to be clearer about the purpose of their engagement efforts, both the engagement outcomes being sought and their understanding of how engagement activities will contribute to these outcomes.	• Being explicit about the purposes and anticipated outcomes of engagement strategies can inform planning and evaluation.
**Clarity around ‘terms of engagement’ for research stakeholders**: researchers and engagement practitioners need to be clearer about the degree of stakeholder decision rights and control throughout the research process, and the processes of stakeholder representation that will allow clear lines of accountability.	• Clearly distinguishing decision rights around the focus, design or implementation of particular research studies, and where there is scope to inform wider institutional policy may help to manage expectations of local research stakeholders
**Early engagement to develop relationships and negotiate engagement** ** processes**: ideally both the purposes and terms of engagement will be negotiated with stakeholders. This means investing the time, space and resources to identify relevant stakeholders, and build the relationships and rapport necessary to be able to productively discuss and agree research purposes and terms of engagement. This may need to be an iterative process and develop over time.	• An inception phase for research should focus on understanding how research affects local stakeholders and on general relationship building • Rather than seeing particular engagement activities as a technical input, engagement should focus on development and monitoring of relationships over time through a range of engagement activities combined and adapted as appropriate • The time and opportunity costs involved for community members engaging with research need to be carefully considered
**Emphasis on researcher responsiveness as well as provision of accurate** ** information on research**: in engagement strategies, structured methods for listening to and responding to research stakeholder concerns may be at least as important as provision of accurate and appropriately tailored information and communication about research.	• Engagement activities should prioritise methods for listening and responding to stakeholder concerns - including formative research, dedicated spaces for raising concerns, and structured feedback from local research staff - with information provision incorporated into these processes.
Research Centres	
**Engagement at research programme level**: programme-wide engagement not linked to particular research studies can support the relationship building that underpins effective engagement, and provide opportunities for community and stakeholder input into research and engagement related policy and practice over time, including around fair benefits for research stakeholders at the individual, community and health infrastructure level. Research centres should be more explicit about the purposes and opportunities of such engagement.	• Research institutions should invest in programme wide engagement not linked to individual research studies and be explicit about the purposes of providing opportunities for relationship building and stakeholder input
**Institutional support for local research and engagement staff**: the complex and demanding roles for research fieldworkers and engagement staff need greater recognition and requisite training, support and supervision. Research institutions policies should reflect commitment to resourcing and developing engagement capacity. Integrating engagement more thoroughly into research programme management also provides the scope for responsive research implementation.	• Experienced based training and supportive supervision for research fieldworkers at the research institution level can help them navigate some of the relational complexities of engagement • Professionalisation of frontline research and engagement staff roles with related career pathways is part of building engagement capacity • Dedicated work with researchers on their understandings and attitudes to CE at the institutional level
**Input on research priorities**: research designs may put limits on how and where research protocols can be influenced, but it is often possible to consult research stakeholders on aspects of research protocols. At the same time, there can be consultation on the thematic focus of research, and the role of responsive research designs, and there is potential to link to broader processes of public consultation on research priorities as part of national research agenda setting and initiatives (like COHRED’s research fairness initiative or the European Responsible Research and Innovation RRI initiative) ^2^.	• Research priorities and designs should be framed in relation to national research agendas and opportunities for stakeholder input linked to broader public consultation on such agendas
**Broader political and ethical focus for health research engagement**: CE processes may be unable to fully address the differences of wealth and power between researchers and local research stakeholders, but can take considered concrete steps to mitigate them. There may also be scope to better integrate individual health studies with wider efforts to strengthen health systems, and enhance health equity (see below).	• Compilation of existing guidance and case studies on provision of individual and community level research benefits could inform careful consideration of the opportunity costs for local research stakeholders for particular studies • Clear procedural standards for community engagement may help monitor levels of meaningful input and control on the part of research stakeholders • Guidance for Ethical Review Board processes could explicitly address the influence of wider structural factors and draw on principles of social justice
Funders and policy makers	
**Greater integration of health research with LMIC health systems:** research institutions and initiatives often bring considerable wealth and resources to LMIC settings where health systems are underdeveloped, with impacts on health care infrastructure and personnel, levels of health care provision, and health surveillance capacity. Further attention could be given to how the health research enterprise links to health system strengthening, and more explicit ways of monitoring this relationship, which may also contribute to improved ‘host country ownership’. Such an integration could strengthen the social value of research overall, and help provide a further rationale for more institutional support for engagement infrastructure and capacity in research centres.	• Plan health research in a way that carefully considers opportunities for strengthening local health systems and minimises any potential harms, and monitors this relationship • Greater attention to policy engagement by both research institutions and funder could help with the above integration • Core funding for health research centres over the long term facilitates programme wide engagement and integration of research plans with local health facility strengthening ^3^
**Greater role for social science research on health and health systems to ** **complement and inform biomedical research:** social science and empirical ethics research are important for understanding prevailing health beliefs and practices and the social and cultural context. It is also important for understanding the contours of health systems and how the international governance of health and health research limits or facilitates CE. In addition, social science expertise can help understand the relationship dynamics that engagement interventions seek to facilitate, and to support their design and evaluation. Recent work on the social determinants of health and health inequalities points to the intimate relationship between the environments in which people grow, live and work, inequalities of wealth and health, the degree of control people have over the fabric of their work and civic lives, and ultimate health outcomes. Greater investment in this broader health research agenda would help to maximise the gains from biomedical research.	• Greater investment in applied social science studies to complement the biomedical research agenda should include research on relational dynamics of engagement, anthropology of health and health systems, the social determinants of health, and international health and research governance • Support the strengthening of the evidence on CE through dedicated resources for formative research, evaluation or implementation studies to accompany biomedical health research
**Inception phases and flexibility in research funding**: the importance of relationship development and early engagement identified above suggest a need for structured inception phases for partnership development and exploration of health research priorities, with funding underpinning them. There also needs to be scope for research budgets and protocols to accommodate stakeholder insights before they are finalized in a way that may preclude further input.	• Funders should explicitly consider processes to allow for budget and protocol flexibility in response to CE and in which circumstances these apply (such as IDRC’s Research Quality Plus tool) ^4^ • Funded inception phases of research can facilitate partnership development and consultation on research focus and design (such as in NIHR Global Health Research Grants) ^5^

## Conclusions

Many of the issues emerging from our analysis converge on the relationship between CE with health research and the underdeveloped health systems in LMICs, and the political and economic factors influencing both. Debates around the scope of health research ethics echo earlier debates around vertical and horizontal approaches to health, and in particular, the role of community participation and decision-making as an integral part of health and wellbeing rather than more narrowly conceived medical health ( Packard, 2000).

Our analysis also resonates with enduring debates around power and participation in health and health research, which have been given new impetus by the recent debates on decolonising global health, including issues such as who controls research agendas and resources; what power relationships frame and imbue the research enterprise; hierarchies of evidence and research methodologies; histories of colonial exploitation; the current politics and economics of global health governance; and the dominance of Northern epistemological and ethical frameworks at the expense of other ways of knowing and understanding the world ( De la Cadena & Blaser, 2018; Fricker, 2007; Packard, 2000; Tuhiwai Smith, 2021).

Ultimately, our analysis suggests that the very relational work that enables the development of ‘working relationships’ between researchers and other local research stakeholders and supports the implementation of research trials also rests on aspects of the prevailing dominant health research paradigm that are problematic ethically. The differences of wealth and power marking the research setting, and the differences of control and power in the ‘terms of engagement’ around research processes tend to be accommodated often with misgivings by local research stakeholders, since the access to valued health care is of primary importance, even if it is not their only concern.

Taking this finding seriously suggests that more attention could be given to how the resources accompanying health research trials - which can include the strengthening of local health infrastructure, staffing, surveillance, diagnostics and supplies - can potentially strengthen health systems. This could be reflected in more explicit planning and monitoring of this relationship ( Asante *et al.*, 2016; Kingori, 2015; Ward *et al.*, 2018) so that health research maximises the opportunities to strengthen local health systems and minimizes the danger of undermining them. At the same time, the relationships that CE facilitates provide an opportunity to mitigate the ethical challenges of the current dominant research paradigm; by building upon and strengthening their collaborative character further to broker better integration of health research and health systems strengthening. Looking beyond biomedical health research to other research paradigms, and considering the role of CE practices in the context of a wider imperative to strengthen health responses and health systems through better research, there is scope to build a more coherent and consistent field of theory and practice around CE.

## Data availability

### Underlying data

All data underlying the results are available as part of the article and no additional source data are required.

### Extended data

Harvard Dataverse: Working relationships across difference – a realist review of community engagement with malaria research. https://doi.org/10.7910/DVN/QVJI3M ( Vincent, 2021).

This project contains the following extended data:

Appendix 1 – Steps in the Realist Review Process

Appendix 2 – Search Strategies

Appendix 3 – Characteristics of included documents including contribution to CMOCs

Appendix 4 – Conceptual resources drawn on in the analysis

Appendix 5 – Full table of CMOCs developed in the analysis

Data are available under the terms of the Creative Commons Zero "No rights reserved" data waiver (CC0 1.0 Public domain dedication).
