# Supplementary material for: ‘Working relationships’ across difference - a realist review of community engagement with malaria research
Source: Wellcome Open Res. 2022 Jan 13;7:13. [Version 1] doi: 10.12688/wellcomeopenres.17192.1 (PMC10444998; doi:10.12688/wellcomeopenres.17192.1)
Supplement: Peer review history

# Reviewer report: Reviewer response for version 1

**DOI:** 10.21956/wellcomeopenres.18995.r65405

**Published:** 2023-09-13

**Authors:** Lucas Tina, Michal Mbinji

**Affiliations:** 1 Kenya Medical Research Institute, Nairobi, Kenya

The paper is well-written and detailed. It addresses an important topic relevant to both qualitative and quantitative research. Some of the important aspects of community engagement (CE) brought out in the paper include the ethical challenges, the resource constraints for CE, the imbalance of power between the participating communities and the research institutions and also the limitations faced by researchers in LMICs when setting the research agenda. Overall, insightful and relevant.

Title: Should it be ‘with malaria’ research or ‘in malaria’ research?

Methodology: For biomedical researchers who may not be very familiar with qualitative research methods, would it be better to detail how/where the 28 papers were “identified’ from? The real search process in Figure 1 was not clear in terms of the flow as you have 2 sets of 28 papers from scoping and from ethics guidance. Were they the same or separate?

Results: The results are well presented and detailed enough to draw the conclusions.

Discussion: The discussion is rich and robust based on the evidence that has been presented.

Conclusion: The conclusion probably should be based on your overall interpretation and less of a repetition of other viewpoints with citations.

 Thank you for your insights and contribution to research regarding this important topic.

Is the review written in accessible language?

Yes

Are all factual statements correct and adequately supported by citations?

Yes

Are the conclusions drawn appropriate in the context of the current research literature?

Yes

Is the topic of the review discussed comprehensively in the context of the current literature?

Yes

Reviewer Expertise:

Clinical Research; Public Health; Malaria Research

We confirm that we have read this submission and believe that we have an appropriate level of expertise to confirm that it is of an acceptable scientific standard.

# Reviewer report: Reviewer response for version 1

**DOI:** 10.21956/wellcomeopenres.18995.r63803

**Published:** 2023-08-22

**Authors:** Justine E Leavy

**Affiliations:** 1 Curtin University, Perth, Western Australia, Australia

**General comments**: The authors have examined Community Engagement (CE) as an increasingly used approach to health research and ethical practice. Whilst LMICS in the past decade have increasingly described using CE in malaria prevention, control and treatment, the evidence base on how CE works is somewhat lacking. The paper is well-written and well-presented and aligns with the RAMESES guidelines for a realist review. I have some minor suggestions outlined below for the authors. Thank you for this approach to curating and publishing the CE findings.

**Introduction: **Community engagement as an integral component of a community-facing health research is well described and the context provided for the paper. The inclusion of the community voice and consumer participation in ethical research is posited (page 3, para 1) and this is a good addition to the discussion around community engagement approaches and aligns with contemporary public health.

 I am interested to see a biomedical approach as the entry point, as public health has embraced community engagement across a range of health issues. I would be keen to have a sentence added to the paper that justifies this approach versus going back to the protocol paper to review how the authors discerned this entry point.

 Page 4, para 3 would the authors consider adding the reference(s) here to support the realist review of CE "...theory driven approach to evidence synthesis”, they have taken?

**Methods**: These align with the RAMESES guidelines and are clearly presented in Table 1 and Figure 1. In text or in Table 1 could you indicate which databases you searched and n = databases you searched? That would save having to go through Appendix 2 and might be of interest to the reader of this paper. The methods are brief and link with App 1 and 2.

 Is the coding framework (Table 1) available for review?

**Results: **These are aligned with your 12 CMOCs and include a narrative summary plus some supporting quotes. I am not sure why you have chosen the ad hoc qualitative comments to cite. This section is quite long, and I wonder if times you need to cite the studies you are highlighting in the results, versus the inclusion of quotes. This may be stylistic, as you then discuss and cite the studies in the Discussion. However, for consideration below are some examples whereby you are stating results but no references are in the text to direct the reader to:

- Page 9, column 2, para 4 you state “In a number of studies involving children (refs go here)…..”
- Page 9, column 2, para 5 “In  a few instances (refs go here) resources were reportedly brokered `by intermediaries…..”
- Page 11, column 2, para 1 “A small proportion of the data in the literature (refs go here)  searched came from primary health care….”
- Page 11, column 2, para 2 “ ……largely descriptive and, with one exception (ref here).”

**Discussion:** This is a robust discussion and brings together your results and the contemporary literature. It is well written and as you note has depth and breadth.

 I enjoyed the discussion around ethics and LMICS. You have mentioned public health and the debates around social, political and economic factors with social justice core to ethical research practice.

 As you have noted the bioethics focus may have limited the number of papers that have community engagement and participatory action research as a central outcome. This is a limitation of the paper.

 Page 16 of 24 column 2, para 3. I wonder if the authors would consider adding some of the content here around the team and their expertise to the Methods and including a section on the research team. We often do this in a qual paper so the reader can get an idea of the research team/authors and any mental biases we may bring to the analysis.

 See: Abercromby *et al.* (2022 ^1^) and Awasthi *et al. *(2022 ^2^).

 Recommendations: The table is a very good addition. Thank you. The paper is long, would the authors consider removing some of the descriptive text here and directing the reader to Table 3?

**Conclusion:** the concluding comments bring the paper together. This is again stylistic, the conclusion should not bring new ideas to the paper, it should conclude what you have found so I was unsure why refs are cited here.

 Thank you for the contribution to the literature.

Is the review written in accessible language?

Yes

Are all factual statements correct and adequately supported by citations?

Partly

Are the conclusions drawn appropriate in the context of the current research literature?

Yes

Is the topic of the review discussed comprehensively in the context of the current literature?

Yes

Reviewer Expertise:

Public Health Evaluation research including injury prevention, physical activity promotion, malaria prevention, knowledge translation and ethics in health promotion.

I confirm that I have read this submission and believe that I have an appropriate level of expertise to confirm that it is of an acceptable scientific standard.

## References

[1] : "I never had a thought about drowning". Exploring water safety attitudes and practices among older adults in Western Australia. *Health Promot J Austr* .2022;33(2) : 10.1002/hpja.522 524-532 10.1002/hpja.522 34278642

[2] : A qualitative study of knowledge, attitudes and perceptions towards malaria prevention among people living in rural upper river valleys of Nepal. *PLoS One* .2022;17(3) : 10.1371/journal.pone.0265561 e0265561 10.1371/journal.pone.0265561 35303022PMC8932613

# Reviewer report: Reviewer response for version 1

**DOI:** 10.21956/wellcomeopenres.18995.r48033

**Published:** 2022-03-07

**Authors:** Bruno Marchal

**Affiliations:** 1 Department of Public Health, Institute of Tropical Medicine Antwerp, Antwerp, Belgium

This paper discusses the relevant topic of community engagement. Based on a good overview of the existing literature, it introduces the conceptual and other problems that may affect a literature review of community engagement. The authors usefully adopt a complexity perspective on community engagement and the choice of the review method (i.e. realist synthesis) is appropriate.

 The authors discuss the definitions of some key terms and refer to the published protocol and build upon the initial programme theory. The results section is clearly and coherently structured around the 4 themes of their initial programme theory and the corresponding CMOCs.

 The discussion section is very rich and positions the findings of the review within the literature.

Is the review written in accessible language?

Yes

Are all factual statements correct and adequately supported by citations?

Yes

Are the conclusions drawn appropriate in the context of the current research literature?

Yes

Is the topic of the review discussed comprehensively in the context of the current literature?

Yes

Reviewer Expertise:

Realist evaluation; health systems and health policy research; complexity

I confirm that I have read this submission and believe that I have an appropriate level of expertise to confirm that it is of an acceptable scientific standard.
